# Monthly Summary

**Published:** 1890-07

**Authors:** 


### Monthly Summary.

A CHESHIRE FARMER AND HIS FALSE TEETH.—A case was heard at Crewe County Court on April 9th, before his Honor Judge Hughes, in which the plaintiff was Charles Edwards, farmer, of Nantwich, who sought to recover £20 from Albert Maurice, dentist, of Liverpool, for the detention of a set of teeth. The plaintiff's evidence was that several years ago he bought a set of teeth for £15. The teeth were plated with gold and encased in vulcanite. A few months ago he went to the defendant and requested him to make some alterations in the upper case so as to make the teeth more comfortable. The alterations were made, and afterwards, at plaintiff's suggestion, the lower set was repaired. Subsequently, while sitting in the defendant's room, the plaintiff suddenly jumped up and demanded an explanation, stating that he had not received back his right set of teeth—that while his teeth were set in a gold plate and cost £15, the set produced were largely vulcanite, and there was not a pound's worth of gold on the case. There was a scene in the defendant's office, and because the plaintiff declined to leave the defendant walked out. The plaintiff's wife and daughter described the original set of teeth with the gold plate. The defendant said that the gold on the old case had been placed on the new, and while he could not swear it was the same piece of gold, he believed it was. A man named Rigby, who modelled the case, said it was the same piece of gold, and that none of it had been taken away. The judge said that the action suggested a serious accusation against the defendant, for which there was no ground. He gave judgment for the defendant with costs.—*Dental Record.*

---

DENTISTRY IN 1892.—In looking over the *Phrenological Journal* for June, 1890, I came across a clever article entitled "Now and then," by L. A. R., giving one a peep into the life of a physician in the years 1822 and 1890. The writer, standing in front of a window in one of our uptown streets, in watching a physician's turnout which has just driven up,—a very stylish coupe, a fine pair of horses with gold-mounted harness, and all in charge of a driver in livery,—recalls to mind a little country town in the Connecticut valley where, during a visit a short while ago to one of the oldest houses in the village, while rumaging in long-neglected corners, found an old manuscript which seemed such a curiosity. It was a statement of account rendered by the village doctor to one of the first families (father, mother and four children), for medical attendance and medicines. An examination will show the difference between now and then. The amount for the first year, was 56 cents: 50 cents for emetic, and 6 cents for eye-water—not a very exorbitant price for medicines. The next year was but a single item—2 visits and 1 emetic, 50 cents. Think of a doctor only charging 25 cents for a visit!

The science of dentistry in those days had not so nearly reached the art of perfection as now, and the country doctor was expected to do all there was to be done in that line, which was confined to the removing of aching molars and an occasional cleaning of the teeth. The account for 1882 included a little in the art of dentistry, *i. e.*, extracting two teeth for Betsy, 17 cents. This looks almost as absurd as some of the prices we see in the stores nowadays. What sort of figure would gentlemen of the dental profession cut on those prices to-day?—*I. S. in Dental Mirror.*

